# Toll-Like Receptor 4 Signaling and Drug Addiction

**DOI:** 10.3389/fphar.2020.603445

**Published:** 2020-11-24

**Authors:** Ruyan Wu, Jun-Xu Li

**Affiliations:** ^1^ School of Medicine, Yangzhou University, Yangzhou, China; ^2^ Department of Pharmacology and Toxicology, University at Buffalo, Buffalo, NY, United States

**Keywords:** toll-like receptor 4, opioids, alcohol, psychostimulants, drug reward, reinstatement, withdrawal

## Abstract

The emphasis of neuronal alterations and adaptations have long been the main focus of the studies of the mechanistic underpinnings of drug addiction. Recent studies have begun to appreciate the role of innate immune system, especially toll-like receptor 4 (TLR4) signaling in drug reward-associated behaviors and physiology. Drugs like opioids, alcohol and psychostimulants activate TLR4 signaling and subsequently induce proinflammatory responses, which in turn contributes to the development of drug addiction. Inhibition of TLR4 or its downstream effectors attenuated the reinforcing effects of opioids, alcohol and psychostimulants, and this effect is also involved in the withdrawal and relapse-like behaviors of different drug classes. However, conflicting results also argue that TLR4-related immune response may play a minimal part in drug addiction. This review discussed the preclinical evidence that whether TLR4 signaling is involved in multiple drug classes action and the possible mechanisms underlying this effect. Moreover, clinical studies which examined the potential efficacy of immune-base pharmacotherapies in treating drug addiction are also discussed.

## Introduction

Neuronal alterations and adaptations have long been the main focus of the studies of the mechanistic underpinnings of drug addiction (Kalivas and O'Brien, 2008; Otis and Mueller, 2017). The emphasis of dopaminergic and glutamatergic signaling in brain reward circuits yield extensive important progress in the study of drug addiction (Sesack and Grace, 2010; Lee et al., 2013; Ma et al., 2014; Zhang et al., 2016). However, they ignore the potential contributions of non-neuronal cells (e.g., microglia and astrocytes) to the synaptic and behavioral adaptations underlying addiction-like behaviors (Kashima and Grueter, 2017). Recent studies have begun to illustrate the role of innate immune system, especially toll-like receptor 4 (TLR4) in drug reward associated behaviors and physiology (Hutchinson et al., 2012; June et al., 2015; Northcutt et al., 2015). This review will briefly discuss the innate immune system and TLR4 signaling. Different classes of drugs including opioids, alcohol and psychostimulants will be reviewed to discuss whether TLR4 signaling can be used as a potential therapeutic target for the treatment of drug addiction. Furthermore, clinical studies which examined the potential efficacy of immune-base pharmacotherapies in treating drug addiction are also discussed.

## The Toll-Like Receptor 4-Related Immune System and Neuronal Disorders

The innate immune system, an evolutionary defense strategy, has been well characterized (Chaplin, 2010; Yu et al., 2018). TLRs are a group of pattern recognition receptors (PRRs) in the innate immune system which detect and respond not only to exogenous pathogen associated molecular patterns (PAMPs), but also to endogenous danger associated molecular patterns (DAMPs) (Koropatnick et al., 2004; Hennessy et al., 2010; Dunne et al., 2011). Activation of TLRs promotes the maturation of antigen presenting cells, like dendritic cells (DC), which subsequently directs the induction of adaptive immunity (Apetoh et al., 2007; Liu et al., 2010; Gaudino and Kumar, 2019). In this regard, TLR agonists have been studied as vaccine adjuvants for cancer or infectious disease (Caron et al., 2005; Sfondrini et al., 2006; Kronenberger and Zeuzem, 2009). However, considering the fact that activation of TLRs leads to the promotion of inflammatory cytokine production, the inhibitors of TLRs also have significant potential as therapeutic agents for inflammatory disorders, such as rheumatoid arthritis (Klareskog et al., 2004; Feldmann, 2009). As a result, the exploitation of TLRs-based therapeutics may be promising for the treatment of multiple infectious and inflammatory diseases.

**FIGURE 1:**
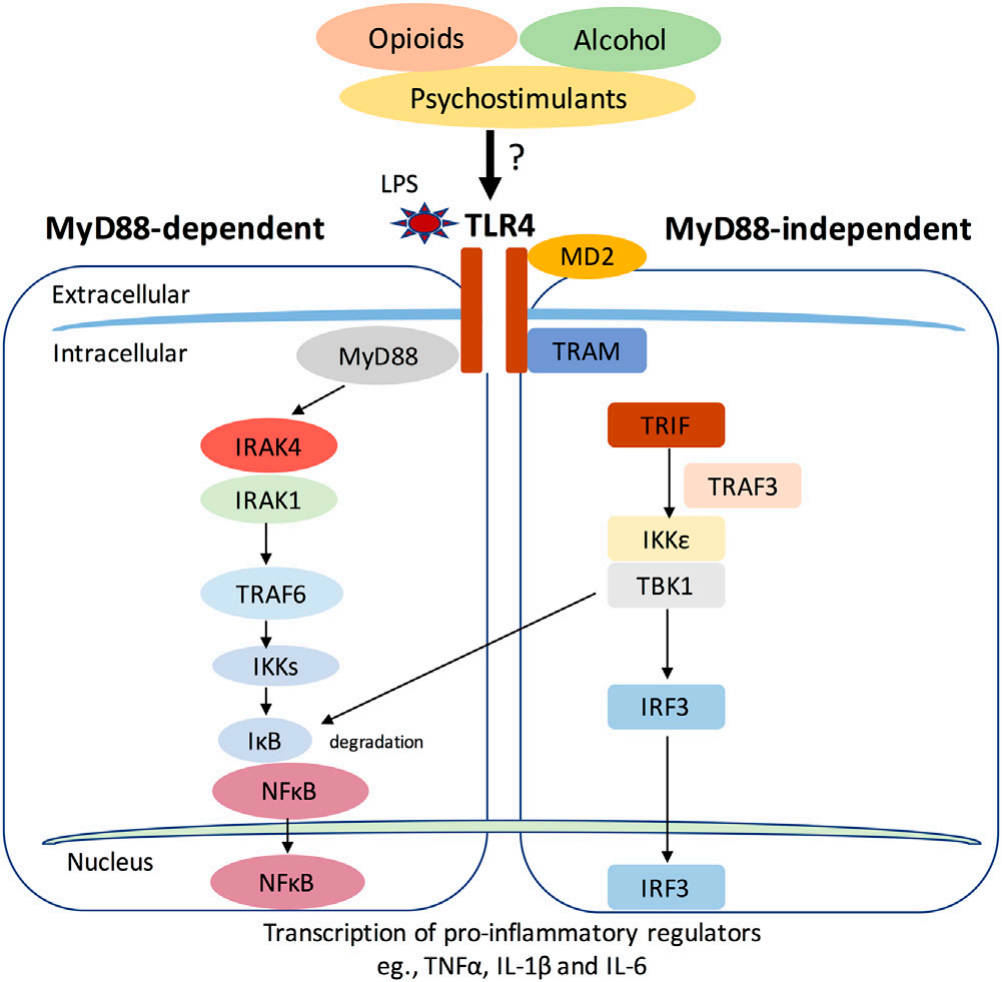
The Toll-like receptor 4 (TLR4) signaling pathway. TLR4 and its co-receptor MD-2 can signal through two different pathways, the myeloid differentiation primary response protein 88 (MyD88)-dependent and MyD88-independent pathway. In MyD88-dependent pathway, the signal transduces through Interleukin 1 receptor associated kinase 4 and 1 (IRAK4 and IRAK1) and the following TNF receptor associated factor 6 (TRAF6). The activation of TRAF6 leads to phosphorylation of inhibitors of nuclear factor κB Kinases (IKKs), which in turn activates the IκB. The activation of IκB leads to its degradation and the initiation of activation of NFκB and the production of proinflammatory cytokines, for example, Tumor Necrosis Factor (TNF), IL-1β and IL-6 (Kawai and Akira, 2007). In contrast, MyD88-independent pathway adopts the adaptor protein TRIF and transduces the signal through TRAF3, TBK1 and IKKε, which then phosphorylates interferon regulatory factor 3 (IRF3). IRF3 then translocates to the nucleus and promotes the transcription of type 1 interferons. Drugs of abuse like opioids, alcohol and psychostimulants may activate TLR4 signaling and induce pro-inflammatory responses.

Apart from their crucial roles in immune system-related diseases, recent studies also suggest that TLRs, especially TLR4, was widely involved in drug addiction-related behaviors (Hennessy et al., 2010; Crews et al., 2017b; Kashima and Grueter, 2017). We will discuss this topic later in detail. In response to pathogen and danger signals, TLR4 and its co-receptor MD-2 can signal through two different pathways, the myeloid differentiation primary response protein 88 (MyD88)-dependent and MyD88-independent pathway (Takeda and Akira, 2004) (Figure 1). In MyD88-dependent pathway, the signal transduces through Interleukin 1 receptor associated kinase 4 and 1 (IRAK4 and IRAK1) and the following TNF receptor associated factor 6 (TRAF6). The activation of TRAF6 leads to phosphorylation of inhibitors of nuclear factor κB Kinases (IKKs), which in turn activates the IκB. The activation of IκB leads to its degradation and the initiation of activation of NFκB and the production of proinflammatory cytokines, for example, Tumor Necrosis Factor (TNF), IL-1β, and IL-6 (Kawai and Akira, 2007). In contrast, MyD88-independent pathway adopts the adaptor protein TRIF and transduces the signal through TRAF3, TBK1, and IKKε, which then phosphorylates interferon regulatory factor 3 (IRF3). IRF3 then translocates to the nucleus and promotes the transcription of type 1 interferons (Takeda and Akira, 2005).

The involvement of TLR4 signaling has been suggested in several neuronal disorders, including neurodegenerative disorders, depression, impulsive behaviors and addiction (Landreth and Reed-Geaghan, 2009; Gesuete et al., 2014; Aurelian et al., 2016; Garcia Bueno et al., 2016; Crews et al., 2017b; Gasiorowski et al., 2018; Nie et al., 2018; Liu et al., 2019). TLR4 is mainly expressed in cells of innate immune system, including microglia and astrocytes (Vaure and Liu, 2014). Consistent to this, several microglia inhibitors attenuated some drug addiction-related behaviors in animal studies. In this review, we will focus on the role of TLR4 in regulating addiction-related behaviors from different drug classes.

## Role of Toll-Like Receptor 4 in Different Classes of Drug Addiction

### Opioids

#### Evidence of a Role of Toll-Like Receptor 4 in Opioid Addiction

It is reported that opioids such as morphine can induce neuroinflammation in the central nervous system (Narita et al., 2006; Yang et al., 2010). Furthermore, this neuroinflammation has been associated with morphine analgesia, dependence, tolerance and withdrawal effects (Eidson and Murphy, 2013; Jacobsen et al., 2014; Mattioli et al., 2014; Eidson et al., 2017; Shah and Choi, 2017). It has been shown that morphine can directly bind to myeloid differentiation protein 2 (MD-2), the accessory receptor of TLR4, and activate TLR4 signaling by inducing the oligomerization of TLR4/MD-2. TLR4/MD-2 knockout animals showed enhanced morphine-induced analgesia, suggesting that blockade of TLR4/MD-2 inhibited morphine-induced proinflammatory responses (Wang et al., 2012b). Inhibition of TLR4 by the levo-isomer of naloxone, (+)-naloxone, attenuated morphine-induced conditioned place preference (CPP). This isomer of naloxone (26.3 mg/kg), which is inactive at opioid receptors, also reduced remifentanil self-administration. Furthermore, genetic knockout of TLR4 or MyD88 decreased oxycodone-induced CPP (Wang et al., 2012b). These results suggest that activation of TLR4 is involved in the rewarding effect of opioids. In addition, *in vivo* microdialysis study showed that (+)-naloxone decreased morphine-induced elevation of dopamine concentration in the shell region of nucleus accumbens (NAc) (Hutchinson et al., 2012). Together, they suggested that the TLR4/MD-2 signaling, along with classic opioid receptors, mediates opioid reward-related behaviors.

However, recent studies which contradict the role of TLR4 in opioid addiction add more complexity to this hypothesis. For example, Phil et al. reported that neither (+)-naloxone nor (+)-naltrexone (3 and 100 μM) inhibit LPS induced TLR4 activation *in vitro* (Skolnick et al., 2014). Stevens et al. also reported that morphine inhibits LPS-induced activation of TLR4 in a concentration-dependent manner, furthermore, this effect was not affected by naltrexone (Stevens et al., 2013). In correspondence of this discrepancy, Watkins lab pointed out the lack of translational potential of (+)-naloxone and (+)-naltrexone from *in vivo* studies considering the lack of biotransformation in in vitro systems (Watkins et al., 2014). In addition, apart from the difference in methodology, they also mentioned that not all agonist-antagonist relationships are equal under different conditions (Watkins et al., 2014). This explanation somewhat makes sense since antagonists could not bind to the receptors if they are fully occupied by respective agonists. Another explanation is that there might be different signaling pathways involved in these interactions thus much effort should be spent to determine the exact signaling underlying the test agents (Watkins et al., 2014). Moreover, mixed results from *in vivo* studies need further consideration. The most intriguing finding is that TLR4 mutant and null mice maintained opioid induced tolerance, hyperalgesia and physical dependence, suggesting a minimal role of TLR4 in opioid actions (Mattioli et al., 2014). Additionally, acute injection of (+)-naltrexone immediately before extinction test had no effect in opioid-seeking behaviors. Meanwhile, acute or chronic delivery of (+)-naltrexone did not affect the extended access heroin self-administration behavior either (Theberge et al., 2013). More importantly, it was found that (+)-naltrexone or (+)-naloxone also reduced food self-administration (Tanda et al., 2016; Yue et al., 2020), suggesting a lack of behavioral specificity of TLR4 antagonists on drug-maintained operant behaviors (Tanda et al., 2016). These results question the validity of TLR4 hypothesis and should be further addressed before it is translated to the clinic. One possible explanation is the selectivity of TLR4 (+)-isomer ligands. Studies have identified non-stereoselective actions of naloxone at sites other than TLR4 (Wang and Burns, 2009; Burns and Wang, 2010; Wang et al., 2012a). Future studies that carefully examine how (+)-isomers act on non-TLR4 are needed to dissect the exact role of TLR4 in opioid addiction.

#### Possible Mechanisms Underlying the Role of Toll-Like Receptor 4 in Opioid Action

Neuronal mechanism of opioid addiction involves the inhibition of GABAergic tone on the mesolimbic dopaminergic reward pathway from ventral tegmental area (VTA) to nucleus accumbens (NAc), resulting in an increase in dopamine release in the NAc (Coller and Hutchinson, 2012; Fields and Margolis, 2015; Volkow and Morales, 2015). Meanwhile, opioids induced glia activation was thought to contribute to their reinforcing and rewarding-like effects, which is achieved possibly through the modulation of TLR4 (Jacobsen et al., 2014). A recent study showed that opioids, like morphine, interacts with MD2 and this binding is TLR4 dependent (Shah et al., 2016). Once activated by opioids, TLR4 increases the levels of pro-inflammatory cytokines and chemokines, which subsequently affect the neuronal transmission and plasticity that is associated with opioid-induced reward (Langlois and Nugent, 2017; Zhang et al., 2020). Therefore, immune factors like TNFα or IL-1β that can modulate synaptic functions may participate in opioid reward. TNFα is a downstream effector of TLR4 signaling and inhibition of TNFα blocked TLR4-mediated morphine-induced neuroinflammation (Kawai and Akira, 2010; Eidson et al., 2017). TNFα is shown to regulate synaptic transmission by affecting the activity of GABA_A_ receptors, AMPA receptors and presynaptic metabotropic glutamate receptors (Bezzi et al., 2001; Stellwagen et al., 2005; Domercq et al., 2006; Stellwagen and Malenka, 2006; Pascual et al., 2012; Lewitus et al., 2014). It may also contribute to opioid reward by altering the opioid sensitivity as shown by a genetic human study (Reyes-Gibby et al., 2008). Like TNFα, activation of TLR4 also leads to an increase in IL-1β expression (Latz et al., 2013). IL-1β mediates long-term potentiation (LTP) which is important to learning and memory (Rizzo et al., 2018). As addiction can be viewed as a type of aberrant reward memory, IL-1β may play a role in the perception of opioid reward (Song et al., 2006). On the other hand, it has been shown that IL-1β suppresses postsynaptic GABA receptor activities through the activation of protein kinase C (PKC) in neurons. Meanwhile, IL-1β inhibits glial glutamate transporter activity, resulting in a deficiency in glutamate supply. This shortage in turn leads to the attenuation of glutamate-glutamine cycle-dependent GABA synthesis. These processes are widely involved in synaptic plasticity which may underlie TLR4-related drug actions (Wang et al., 2000). Though the exact role of TLR4 signaling in mediating opioid addiction remains unclear, it should be noted that activation of TLR4 mediated central immune response by drug of abuse can only work in concert with the well-established neuronal mechanisms of reward, as the central immune signaling alone cannot produce related behavioral effects (Coller and Hutchinson, 2012).

#### Clinical Implications for Toll-Like Receptor 4-Related Innate Immune Modulation in Opioid Addiction

With the knowledge that TLR4-related glial activity may play a role in opioid addiction preclinically, exploration of novel pharmacological treatments for opioid addiction by targeting the glial activity remains a promising choice (Zhang et al., 2020). One of the most studied existing medication is ibudilast, which is a non-selective phosphodiesterase inhibitor and TLR4 antagonist (Ruiz-Perez et al., 2016). Ibudilast is widely used in Asia for the treatment of asthma and post-stroke dizziness (Gibson et al., 2006), which showed a well safety and tolerability of a single dose (30 mg) and a 30-mg twice daily 2-week regimen in healthy subjects (Rolan et al., 2008). Recently, Comer lab has carefully examined the potential of ibudilast on opioid-induced analgesia, subjective and withdrawal symptoms in opioid-dependent volunteers. They found that ibudilast (40 mg, bid, 1 week) enhanced the oxycodone-induced analgesia as measured by subjective pain ratings (Cooper et al., 2017). Moreover, volunteers who received ibudilast (20 and 40 mg, bid, 2 weeks) also had lower ratings of withdrawal symptoms (Cooper et al., 2016). However, ibudilast did not affect oxycodone-induced subjective drug effect ratings (e.g., “high, good effect, I would pay”) (Cooper et al., 2017). In contrary, another study by Metz et al. reported that ibudilast decreased the rating of drug like following 15 mg oxycodone in opioid-dependent volunteers (Metz et al., 2017). Ibudilast also significantly decreased the drug breakpoint value under 15 mg oxycodone condition, but not under 30 mg oxycodone condition. They also observed similar results that craving for heroin, cocaine and tobacco was also reduced under active ibudilast compared with placebo (Metz et al., 2017). It seems contradicting on whether ibudilast could decrease the subjective and reinforcing effects based on these results. However, they may reconcile at some point since Metz et al. found ibudilast reduced the craving following 15 mg, but not 30 mg oxycodone, while Comer lab examined higher doses of opioid (e.g., 30 mg morphine or 25, 50 mg/70 kg oxycodone). The discrepancy may also attribute to the limited sample volume and too few trails (Zhang et al., 2020). Meanwhile, considerable individual variability may also add up to weaken the power of these studies. Therefore, future investigations with increased sample size are urgently needed to verify the clinical potential of glia modulators on opioid addiction.

Despite the clinical results of ibudilast are conflicting, the therapeutic potential of glial modulators in preventing opioid abuse should not be underestimated. Currently, other glial modulators are being examined for their ability in treating opioid use disorders as well. For example, minocycline increased accuracy on a cognition task in individuals with opioid use disorder, suggesting an effect like cognition enhancement. However, the pain threshold or tolerance, opioid craving and withdrawal weren’t changed by minocycline treatment (Arout et al., 2019). Another glial modulator, cannabidiol, was shown to decrease opioid-induced craving and anxiety in drug-abstinent individuals with opioid use disorder (Hurd et al., 2015; Hurd et al., 2019). These results, albeit complex, suggesting a promising role of glial modulators in treating opioid addiction. Further studies with more dose regimen, greater sample size and prolonged trials are needed to figure out their exact roles.

### Alcohol

#### Evidence of a Role of Toll-Like Receptor 4 in Alcohol Addiction

Studies have suggested that TLR4 affects some behavioral effects of ethanol (Pandey, 2012; Wu et al., 2012; Pascual et al., 2015; Blednov et al., 2017a). Both pharmacological inhibition of TLR4 and genetic deficiency of TLR4 or MyD88 significantly decreased the duration of loss of righting reflex (LORR) and reduced recovery time in motor impairment (rotarod test). Importantly, these effects were not due to changes of ethanol pharmacokinetics (Wu et al., 2012; Blednov et al., 2017a). In addition, TLR4-deficient mice showed lower sensitivity to pentobarbital-induced sedative effect and faster recovery from diazepam-induced motor impairment, suggesting a crosstalk between TLR4 and GABAergic functions (Blednov et al., 2017a). Chronic exposure of ethanol increases the expression of many cytokines (TNF-α, IL-1β) and chemokines (CX_3_CL1, MCP-1) in the mice striatum and serum (Pascual et al., 2015). Interestingly, mice lacking TLR4 or TLR2 receptors are protected against ethanol-induced cytokine release (Pascual et al., 2015). These mice also showed less ethanol abstinence-induced behavioral changes such as increased anxiety (Pascual et al., 2015). Combined, these results suggest a clear involvement of TLR4 signaling in some acute and chronic effects of ethanol.

Binge drinking represents the initial stage of alcohol addiction, which has a link with anxiety (Chikritzhs et al., 2001; Naimi et al., 2003; Edenberg et al., 2004; Ducci et al., 2007). It is also suggested that TLR4-GABAA α2 subunit pathway regulates alcohol binge drinking in rodents (Spanagel et al., 1995; Roberts et al., 1996; Foster et al., 2004). By infusing a GABAA α2 siRNA vector in central nucleus of amygdala (CeA) of alcohol-preferring rats (P rats), Juan et al. reported a significant and specific reduction of alcohol binge drinking, reduced α2 subtype GABAA receptor expression, decreased GABAA receptor density and inhibition of TLR4(Liu et al., 2011). Moreover, TLR4 siRNA infusion to the CeA also decreased binge drinking behaviors without affecting sucrose intake, suggesting a specificity on alcohol-related behaviors (Liu et al., 2011). Similarly, another study showed that TLR4 or MCP-1 siRNA in CeA or VTA of P rats decreased the corresponding gene expression and binge drinking behavior (June et al., 2015). A further study also showed that α2 subtype of GABAA receptor activates TLR4 signals in neurons in VTA (Balan et al., 2018). Studies from inhibitors also support the central role of TLR4 in binge drinking as TLR4 inhibitor T5342126 decreased ethanol drinking (Bajo et al., 2016).

However, a recent comprehensive study has shown that manipulations of TLR4 may have minimal impact on excessive ethanol drinking behavior (Harris et al., 2017). By using the multiple models: TLR4-KO rats, selective Tlr4 knockdown in mouse NAc and inhibitor (+)-naloxone in different species, Harris et al. demonstrated that either genetic deletion of TLR4 or pharmacological inhibition of TLR4 or Tlr4 knockdown did not affect alcohol intake using two-bottle choice procedure and drinking-in-the-dark assay (Harris et al., 2017). Meanwhile, specific Tlr4 knockdown in mouse NAc did not alter ethanol intake and preference for ethanol in the 24 h continuous access two-bottle test. These results suggest that TLR4 was not important to the excessive drinking behavior and subsequently question the hypothesis that TLR4 is a critical component in mediating alcohol response. One explanation is that Harris et al. examined a Tlr4 knockdown in the NAc while previous studies tested in the CeA or VTA. The difference in brain regions tested may lead to discrepancy in findings. In addition, while Harris used two-bottle and drinking-in-the dark tests, previous studies adopted binge-drinking model in P rats. It seems reasonable that increased GABAergic responses in P rats contribute to their altered binge-drinking behaviors. Nevertheless, they also reported consistent results that TLR4-KO rats had reduced duration of LORR, and CeA deletion of Tlr4 changed GABA_A_ α2 subtype receptor function (Harris et al., 2017). These results at least suggest essential role of TLR4 signaling in mediating acute behavioral effects of ethanol (Alfonso-Loeches et al., 2010; Pascual et al., 2015; Blednov et al., 2017b). More studies are needed to disentangle the exact role of TLR4 signaling in alcohol addiction-related effects.

#### Possible Mechanisms Underlying the Role of Toll-Like Receptor 4 in Alcohol Action

Alcohol intake increases gut permeability, allowing translocation of bacterial toxins like LPS through the intestines into blood stream (Parlesak et al., 2000; Leclercq et al., 2012; Leclercq et al., 2014). LPS in the bloodstream reaches the liver and stimulate TLR4 in liver Kupffer cells, resulting in an increase in pro-inflammatory cytokines and chemokines (Roh and Seki, 2013) which can cross the blood-brain barrier and activate the glia cells in the brain (Montesinos et al., 2016). On the other hand, alcohol can activate glial TLR4 (Blanco et al., 2005; Fernandez-Lizarbe et al., 2009) and induce translocation to the lipid raft and promoting the activation of downstream effectors (Blanco et al., 2005; Blanco et al., 2008; Fernandez-Lizarbe et al., 2009). This activation contributes to ethanol-induced neuroinflammation and neurodegeneration (Alfonso-Loeches et al., 2010; Pascual et al., 2011; Alfonso-Loeches et al., 2012). Both chronic and acute exposure to ethanol cause TLR4-associated signaling response *in vivo* and *in vitro* (Blanco et al., 2004; Valles et al., 2004; Blanco et al., 2005; Blanco and Guerri, 2007). Conversely, inhibition of TLR4 blocks the proinflammatory responses and prevents cell damage (Blanco et al., 2005)*.*

Indeed, the activation of innate immunity and TLR4 signaling appear to be essential for alcohol addiction-like behaviors (Pascual et al., 2011). The persistent activation of neuroinflammation exacerbates the neurodegeneration of key brain regions involved with excessive alcohol consumption, thus underlying at least partly the mechanisms that regulate the development of alcohol addiction (Crews et al., 2015; Flores-Bastias and Karahanian, 2018). Alcohol consumption promotes innate immune activation that are linked to alterations in executive function, reward and negative affect-craving-anxiety that contribute to alcohol use disorders (Vetreno and Crews, 2014; Crews et al., 2017a). Alcohol-induced cell damage in brain regions like prefrontal cortex may cause an executive dysfunction over behavioral inhibition (like binge drinking) and also a lack of inhibition in mesolimbic areas, which is turn increase drinking motivation (Crews et al., 2011; Crews et al., 2015). The loss of control over progression from initial intoxication and binge drinking stage to compulsive drinking stage may lead to the development of alcohol addiction.

#### Clinical Implications for Toll-Like Receptor 4-Related Innate Immune Modulation in Alcohol Addiction

Most recent studies have examined the potential of ibudilast in treating alcohol use disorders (AUD). In a recent randomized, double-blinded and placebo-control study, ibudilast was tested for its safety, tolerability and initial efficacy in mild-severe AUD outpatients (Ray et al., 2017). Ibudilast (50 mg, bid) was well-tolerated with no severe adverse events in the trial. However, ibudilast was not able to affect subjective response to alcohol as shown by craving, stimulation, sedation, positive or negative mood, “like” or “wanting” alcohol (Ray et al., 2017). Nevertheless, ibudilast was associated with mood improvements and decreased tonic level of craving after stress and alcohol cue exposure (Ray et al., 2017). Further analysis revealed that ibudilast attenuated the stimulating and mood-altering effects of alcohol among individuals with higher depressive ratings (Ray et al., 2017). This study suggested a possible mood-modulating effect of ibudilast in treating AUD and which may contribute to the reduced alcohol craving after stress or cue exposure. A more recent study further examined whether ibudilast affect other appetitive behavior, like food craving in AUD participants (Cummings et al., 2018). They found that ibudilast did not affect tonic high-fat/high-sugar food craving, indicating a specificity of modulating drinking behaviors (Cummings et al., 2018). These results provide the first evidence of whether ibudilast could be used for the treatment of alcohol addiction. However, it is still unclear whether ibudilast could decrease the subjective effect or alcohol intake since only few studies have examined this effect with limited participants and trails. More extensive studies are warranted to examine the potential of ibudilast on alcohol intake, withdrawal and relapse. Meanwhile, other neuroimmune modulators, like minocycline, PDE-4 inhibitor apremilast or selective PPARα agonist fenofibrate are undergoing clinical trials to determine their efficacy in reducing alcohol use, craving and related neuroinflammation (Erickson et al., 2019). More importantly, future studies that determine the effect of combination of neuroimmune pharmacotherapies with established medications for alcohol addiction are also warranted (Stopponi et al., 2013).

### Psychostimulants

#### Evidence of a Role of Toll-Like Receptor 4 in Psychostimulants Addiction

There is a large body of literature that psychostimulants like cocaine and methamphetamine can activate and modulate neuroimmune responses (Loftis and Janowsky, 2014; Lacagnina et al., 2017). *In vitro* studies suggest that psychostimulants directly modulate the TLR4 signaling activity. For example, cocaine exposure increases the expression of TLR4 in BV-2 cells in a dose-dependent manner (Periyasamy et al., 2018), and brain TLR4 expression was higher in mice self-administering cocaine than in those self-administering saline (Northcutt et al., 2015; Brown et al., 2018; Periyasamy et al., 2018), indicating an upregulation of TLR4 signaling by cocaine exposure. Similarly, methamphetamine treatment increases the expression of TLR4 in cultured astrocytes (Du et al., 2017) while silencing TLR4 expression using siRNA abolishes methamphetamine-induced expression of IL-1β and IL-18 (Du et al., 2017). *In vivo* evidence are consistent with these findings in that mice pretreated with the TLR4 inhibitor TAK-242 showed significantly decreased expression of IL-1β and IL-18 in striatum induced by methamphetamine (Du et al., 2017). All these results suggest that psychostimulants can activate TLR4 which may contribute to the behavioral effects of the drug.

Pharmacological antagonism of TLR4 also showed consistent results. Indeed, (+)-naloxone blocked cocaine-induced proinflammatory signaling both *in vitro* and *in vivo* (Northcutt et al., 2015). More importantly, evidence showed that TLR4 signaling at least partially contributed to cocaine-induced elevation of NAc dopamine (Northcutt et al., 2015). (+)-Naloxone ameliorated the robust increase in NAc dopamine induced by cocaine while it alone did not produce any effect (Ducci et al., 2007). Conversely, activation of TLR4 in the VTA by local LPS injection was sufficient to produce an elevation of dopamine in the NAc (Nie et al., 2018), suggesting a mediating role of TLR4 in cocaine-induced dopamine release. Behavioral studies further strengthened this notion as pretreatment of (+)-naloxone blocked the development of cocaine CPP and responding for cocaine injection (Northcutt et al., 2015). However, (+)-naloxone did not decrease the responding for food, which suggests that general operant behaviors are intact (Northcutt et al., 2015). In addition, a recent study has shown that TLR4 contributes to the drug-induced reinstatement of cocaine seeking (Brown et al., 2018). Local antagonism of TLR4 in the VTA decreased cocaine-seeking but not sucrose-seeking behavior (Brown et al., 2018). Collectively, these results showed that psychostimulant drugs activate TLR4 signaling which in turn contributes to the reinforcing and relapse-related effects of the drugs.

However, inconsistent evidence exists that pharmacological blockade of TLR4 by (+)-naloxone and (+)-naltrexone which attenuated cocaine self-administration also decreased food-maintained responding, suggesting a non-specific effect (Tanda et al., 2016). Meanwhile, pretreatment with (+)-naloxone and (+)-naltrexone did not affect the increased dopamine levels induced by cocaine (Tanda et al., 2016). Furthermore, a more recent study shows that TNF-α, an inflammatory cytokine downstream TLR4, suppresses cocaine-induced behavioral sensitization by depressing cocaine-induced synaptic changes in NAc core (Lewitus et al., 2016). Indeed, activation of microglia by cocaine increases TNF-α production, which subsequently limits the cocaine-induced changes to NAc circuity, and finally restrains the development of cocaine-induced behavioral sensitization. More importantly, after a period of abstinence, mild activation of TLR4 can reactivate microglia and reduce both synaptic strength in the NAc and locomotor activity to cocaine (Lewitus et al., 2016). Thus, it suggests that augmenting microglia responses through TLR4 or others might be a reasonable approach to treat addiction. Nonetheless, another study showed that TLR4 knockout (KO) mice had a deficit in low-frequency stimulation-induced NMDAR-dependent long-term depression (LTD) in NAc core, which contributed to an attenuation in drug reward learning (Kashima and Grueter, 2017). These mixed results about the role of TLR4 in psychostimulants action make it difficult to draw specific conclusions here. Explanations for this discrepancy may involve differences in addiction-related behaviors and stages of addiction studied. These differences promote continued examination of the effect of TLR4 in drug addiction.

#### Possible Mechanisms Underlying the Role of Toll-Like Receptor 4 in Psychostimulants Action

Studies have shown that cocaine and methamphetamine bind to the accessory receptor of TLR4, MD-2, which stabilizes the conformation of TLR4/MD-2 heterodimers. Methamphetamine binding activates TLR4 and NF-Kβ and upregulates the microglia activation marker CD11b and IL-6 in the VTA, which can be abolished by TLR4 antagonists LPS-RS and TAK-242 (Bachtell et al., 2015; Wang et al., 2019). Meanwhile, the TLR4 antagonist (+)-naloxone or (+)-naltrexone docked to the same pocket of MD-2, competing with other molecules, suggesting a potential modulatory role of TLR4 antagonist in psychostimulants-induced TLR4 activation (Northcutt et al., 2015). TLR4 activation by cocaine or methamphetamine leads to the increased levels of proinflammatory cytokines or chemokines, which subsequently contributes to abnormal neuronal excitatory and toxicity. This non-neuronal mechanism is believed to work in combination with the well-known neuronal circuity, such as psychostimulants-induced alterations of dopamine transporters functions (Hall et al., 2004), to achieve the associated development of drug addiction. It remains unclear how these two mechanisms synergize and result in addiction-like behaviors. However, alterations in synaptic plasticity and neuronal transmission induced by immune response are believed to play a part.

#### Clinical Implications for Toll-Like Receptor 4-Related Innate Immune Modulation in Psychostimulants Addiction

Currently, there are no FDA-approved medications for the treatment of psychostimulants addiction. However, accumulating evidence suggests that targeting neuroinflammation might be a promising strategy for developing a potential pharmacotherapy to treat stimulants addiction. In 2010, a case study reported that minocycline improved the psychotic symptoms in a female patient who had methamphetamine use disorder, suggesting a promising role of minocycline in treating methamphetamine addiction (Tanibuchi et al., 2010). However, there were no follow-up clinical studies which further examined the potential of minocycline in methamphetamine use disorders since then. On the other hand, ibudilast, which has been shown to reduce methamphetamine self-administration and reinstatement in animals, was examined for its efficacy clinically. Ibudilast was able to reduce several methamphetamine–related subjective effects (Worley et al., 2016). Further study also demonstrated that ibudilast may improve attention during early abstinence from methamphetamine dependence (Birath et al., 2017). Despite the limitations of these early-stage studies, they provide first evidence that ibudilast might serve as a potential pharmacotherapy for methamphetamine use disorders. However, a most recent study showed that ibudilast did not affect methamphetamine abstinence (Heinzerling et al., 2020). This randomize trials included 64 participants for ibudilast group and 61 for placebo. Urine specimens for drug screens were collected twice a week. Nonetheless, there was no correlation between serum ibudilast levels and methamphetamine use during treatment. This study suggests that ibudilast might not be able to affect methamphetamine abstinence, yet it’s hard to conclude that ibudilast has no effect on methamphetamine action since no further evidence reported whether ibudilast could affect methamphetamine intake or craving. Actually, a pilot randomized clinical research demonstrated that ibudilast reduced the increased levels of peripheral markers of inflammation induced by methamphetamine treatment in patients, which have implications for the development of treatment for psychostimulants addiction (Li et al., 2020). These results are encouraging, though more studies are needed to examine the long-term effect of ibudilast on both peripheral and central neuroinflammation markers and how these modulations link to clinical outcomes.

## Future Directions

While significant effort has been made to illustrate the role of TLR4-related immune response in drug addiction, it is early to reach a solid conclusion. Since debates are remained about whether TLR4 is essential to drugs of abuse, further studies should further examine the link between TLR4-related immune activation and different stages of drug addiction. Meanwhile, it is generally believed that TLR4-related immune response activated by drugs of abuse work in concert with established neuronal mechanisms, which contribute to the rewarding and reinforcing effects. However, it remains elusive how non-neuronal activation communicates with the mesocorticolimbic reward system which underlies drug addiction-related behaviors. Thus, examinations of the interactions between these two systems would add valuable information to the knowledge of the mechanism underlying drug addiction. More importantly, these studies would further suggest the potential and novel therapeutic targets for the treatments of drug addiction. Moreover, randomized clinical trial which examines the potential efficacy of immune-based pharmacotherapies in drug addiction is in its infancy as conflicting results from clinical data weakens the translational value of the immune-based therapies. Current clinical trials have limited sample size and test restricted time window, dosage effects and drug actions. Consequently, future clinical studies including more participants, examining long-term efficacy and multiple dose-effects of immune-based pharmacotherapies for different stages of drug addiction are warranted.

## Summary

Emerging evidence suggest an important role of the neuroimmune system, especially TLR4, in addiction-related effects of different classes of drugs such as opioids, alcohol and psychostimulants. Drugs of abuse activate TLR4 signaling and the modulation of TLR4 signaling has been shown to be involved in different stages of drug addiction (binge or intoxication, withdrawal and relapse). Accordingly, pharmacological strategies such as non-specific microglia inhibition is a potentially promising approach to treat drug abuse. This is a burgeoning field that requires more mechanistically based studies for target validation and future clinical trials with clinically approved drugs to repurpose for the treatment of drug addiction.

## Author Contributions

RW: conception, design, gathering, interpretation of data and writing; JL: conception, interpretation of data and writing.

## Funding

RW was partially supported by the National Natural Science Foundation of China [Grant 81701340] and Natural Science Foundation of Jiangsu Province [Grant BK 20170517].

## Conflict of Interest

The authors declare that the research was conducted in the absence of any commercial or financial relationships that could be construed as a potential conflict of interest.
